# The prognostic value and immunological role of CD44 in pan-cancer study

**DOI:** 10.1038/s41598-023-34154-3

**Published:** 2023-04-28

**Authors:** Shaoyong Chen, Siqin Zhang, Shaohua Chen, Fei Ma

**Affiliations:** 1 College of Stomatology, Guangxi Medical University, Nanning, Guangxi China; 2 Guangxi Medical University, Nanning, Guangxi China

**Keywords:** Cancer genetics, Cancer microenvironment

## Abstract

To investigate the correlation between cluster of differentiation-44 (CD44) expression and immunotherapy response and identify its possible predictive value in pan-cancer. Datasets of 33 cancer types from The Cancer Genome Atlas (TCGA) database were applied to investigate the relationship of CD44 expression with prognosis, tumor mutational burden (TMB), and microsatellite instability (MSI), and determine its potential prognostic value in pan-cancer. Patients were split into high-risk and low-risk cancer groups based on the survival outcomes of various cancer types. Additionally, the underlying mechanisms of CD44 in the tumor microenvironment (TME) were analyzed using ESTIMATE and CIBERSORT algorithms and Gene Set Enrichment Analysis (GSEA). Subsequently, the biological role of CD44 at single-cell level was investigated using CancerSEA database. Variable expression levels of CD44 between tumor and adjacent normal tissues were identified in pan-cancer datasets, further survival analysis revealed that CD44 expression was associated with multiple clinical annotations and survival indicators. Besides, the expression of CD44 was significantly associated with TMB and MSI in 10 types and 6 types of cancer, respectively, indicating it could be exploited as a potential biomarker predicting immunotherapy outcomes. Meanwhile, CD44 could influence several crucial immune cell-related pathways. and the results revealed by CancerSEA database denoted the correlation of CD44 with malignant phenotype and functional states, further indicating it can serve as a potential therapeutic target in cancer management. Our study demonstrated that CD44 shows great promise as a prognostic biomarker in numerous cancers, which will assist in developing new strategies in cancer management.

## Introduction

Cancer morbidity and mortality are rapidly escalating, especially in developed countries^1^. According to a systematic analysis of 29 cancer types in 195 countries, about 9.6 million individuals died from cancer-related deaths in 2017^2^. Currently, cancer therapies include surgery, chemotherapy, targeted therapy, and radiotherapy. Despite the clinical success of these treatments, the prognosis and tolerability of cancer patients remain unsatisfactory owing to the limited efficacy and side effects of anti-cancer agents^3^. Fortunately, multi-omics has facilitated the molecular characterization of myriads of human diseases^4^, and cancer immunotherapy has revolutionized cancer management following advancements in immunotherapy, presenting encouraging results in clinical trials^5^. However, non-responsiveness to immunotherapy was also observed in specific cancer types and patients, indicating the presence of intrinsic resistance or naturally acquired resistance^6^. Hence, it is of tremendous significance to ascertain novel biomarkers for cancer diagnosis and, most importantly, predict the efficacy of immunotherapy^7,8^.

Cluster of differentiation-44 (CD44) is a member of the non-kinase, single-span transmembrane glycoproteins family, which can contribute to cancer stem cells (CSCs) function and is generally recognized as a molecular marker for CSCs^9^. It has been well documented that CD44 expression in CSCs is associated with metastasis and capacity resisting to apoptosis of cancer cells^10^. Similarly, Zhang et al.^11^ reported that CD44 was related to cellular states and phenotypes of tumor cells in breast cancer, while Gomez et al.^12^ noted that CD44 expression influenced cancer cell plasticity through tumor-associated macrophages (TAM), inferring that CD44 is a surface marker defining Head and neck squamous cell carcinoma (HNSC). The association of CD44 expression with other cancers, including prostate cancer^13^, colon cancer^14^, bladder cancer^15^, and gastric cancer^16^, was also reported, with evidence manifesting that CD44 could promote tumorigenes is and has the potential to be a molecular target in cancer therapy^17^. Notwithstanding, most studies on CD44 were restricted to specific cancer types, obscuring its exact role in tumorigenesis. Thus, it is imperative to deeply explore the role of CD44 using pan-cancer analysis, thereby providing a novel avenue for developing novel treatments and individualized therapies.

The present study systematically assessed the predictive significance of CD44 expression in 33 cancer types using The Cancer Genome Atlas (TCGA) database. Then, the potential correlation of CD44 with tumor mutational burden (TMB) and microsatellite instability (MSI) was evaluated. Moreover, the underlying mechanisms of CD44 were also examined using the ESTIMATE and CIBERSORT algorithms and Gene Set Enrichment Analysis (GSEA). Our pan-cancer investigation aimed to illustrate the association of CD44 with immunotherapy response in oncogenesis among varying cancer types and its potential for predicting prognosis in pan-cancer patients.

## Materials and methods

### CD44 expression analysis in pan-cancer

CD44 expression information, including RNA sequences, somatic mutations, and related clinical annotations for 33 cancers (Table 1), were downloaded from the University of California Santa Cruz (UCSC) Xena database.

**Table 1:** The abbreviation of 33 cancer types from TCGA database.

Cancer types	Abbreviation
Adrenocortical carcinoma	ACC
Bladder urothelial carcinoma	BLCA
Breast invasive carcinoma	BRCA
Cervical squamous cell carcinoma and endocervical adenocarcinoma	CESC
Cholangiocarcinoma	CHOL
Colon adenocarcinoma	COAD
Lymphoid neoplasm diffuse large B-cell lymphoma	DLBC
Esophageal carcinoma	ESCA
Glioblastoma multiforme	GBM
Head and neck squamous cell carcinoma	HNSC
Kidney chromophobe	KICH
Kidney renal clear cell carcinoma	KIRC
Kidney renal papillary cell carcinoma	KIRP
Acute myeloid leukemia	LAML
Brain lower grade glioma	LGG
Liver hepatocellular carcinoma	LIHC
Lung adenocarcinoma	LUAD
Lung squamous cell carcinoma	LUSC
Mesothelioma	MESO
Ovarian serous cystadenocarcinoma	OV
Pancreatic adenocarcinoma	PAAD
Pheochromocytoma and paraganglioma	PCPG
Prostate adenocarcinoma	PRAD
Rectum adenocarcinoma	READ
Sarcoma	SARC
Skin cutaneous melanoma	SKCM
Stomach adenocarcinoma	STAD
Testicular germ cell tumors	TGCT
Thyroid carcinoma	THCA
Thymoma	THYM
Uterine corpus endometrial carcinoma	UCEC
Uterine carcinosarcoma	UCS
Uveal melanoma	UVM

Afterward, CD44 expression was analyzed through the Tumor IMmune Estimation Resource (TIMER) database. The gene expression levels were normalized using log2 conversion. Meanwhile, R-package “ggpubr” was employed to visualize the results obtained from the TCGA database.

### Association analyses between CD44 and survival data

Survival information for each sample was retrieved from the TCGA database, and the correlation of CD44 expression with survival indicators, including overall survival (OS), progression-free interval (PFI), disease-free interval (DFI), and disease-specific survival (DSS), were examined by the Kaplan–Meier method. Patients of varying cancer types were divided into two groups (high-risk and low-risk groups) using the median CD44 expression level as the cut-off value. The above analyses were performed using the R packages “survminer” and “survival”. In addition, univariate Cox analysis was used to score the correlation between CD44 expression and the survival indicators using the R package “survival”, and visualized by the R package “forestplot”.

### Biomarker analysis of CD44 as an indicator of response to targeted therapy

The receiver operating characteristic (ROC) plotter database^18,19^ was used to assess the feasibility of CD44 in targeted therapy responses to various cancer cohorts such as breast carcinoma (BRCA) treated with anti-HER2 antibody, colorectal carcinoma treated with bevacizumab, and ovarian cancer cohorts treated with targeted therapy. The ROC curves were used to present predictive ability of CD44 in predicting the efficacy of various antitumor agents in the above analysis.

### Association between the expression of CD44 and clinical annotations, TMB and MSI

Clinical annotations (age, gender, and tumor stage) of pan-cancer patients were downloaded from the TCGA database, and Spearman correlation analysis between CD44 expression and clinical annotations was performed by the R packages “limma” and “ggpubr”. The correlation of CD44 expression with MSI or TMB was analyzed using Spearman analysis and visualized by radar plots through the R package “fmsb”. Next, Tumor Immune Dysfunction and Exclusion (TIDE) database was used to assess the potential of CD44 as a responsive biomarker for the cancer cohorts treated with immunotherapy (http://tide.dfci.harvard.edu/), which is a web application integrating the expression profiles of T cell dysfunction and exclusion, thereby modeling immune evasion of tumor cells, and has the potential to predict the response of immune checkpoint blockade (ICB)^20^.

### Relationship between CD44 expression, immune components, and tumor-infiltrating immune cell profiles

To determine the proportion of immune and stromal components in the tumor microenvironment (TME), the ESTIMATE algorithm was applied to evaluate the associations between the immune and stromal scores with CD44 expression levels through R packages “estimate” and “limma”. Next, relative tumor-infiltrating immune cells (TICs) levels were calculated using the CIBERSORT algorithm, and samples of the tumor with *P* < 0.001 were retained for subsequent evaluations. Correlation analysis between CD44 expression and relative TICs levels was conducted using the R packages “ggplot2”, “ggpubr”, and “ggExtra”.

### Single-cell analysis for CD44

Cancer single-cell state atlas (CancerSEA) (http://biocc.hrbmu.edu.cn/CancerSEA/) is the dedicated database to explore the distinct functional states of different cancer cells at single-cell resolutions^21^. We used the CancerSEA database to evaluate the functional role of CD44.

### Immune-related genes and enrichment analysis in various cancer types

The R package “limma” was employed to perform co-expression analysis between CD44 and immune-related genes, and the results were visualized by “reshape2” and “RColorBrewer” R packages. Then, GSEA analysis, including Gene Ontology (GO) and Kyoto Encyclopedia of Genes and Genomes (KEGG) analyses (www.kegg.jp/kegg/kegg1.html)^22^, was carried out to explore the role of CD44 in pan-cancers, and the top five enrichment terms of each tumor type were illustrated using the R package “ClusterProfiler”.

### Statistical analysis

Alterations of CD44 expression in tumor tissues and normal tissues were estimated using the Wilcoxon test. In survival analysis, the relationship between CD44 expression and survival information in pan-cancer patients was determined using Kaplan–Meier and univariate Cox regression analyses. The evaluation of these data was conducted through R software (Version 4.0.3) and Strawberry Perl (Version 5.30.0.1). *P* < 0.005 was considered statistically significant^23^.

### Ethical approval and consent to participate

Our study did not require ethical board approval because it did not contain human or animal trials.

## Results

### Pan-cancer expression profiles of CD44

**Figure 1:**
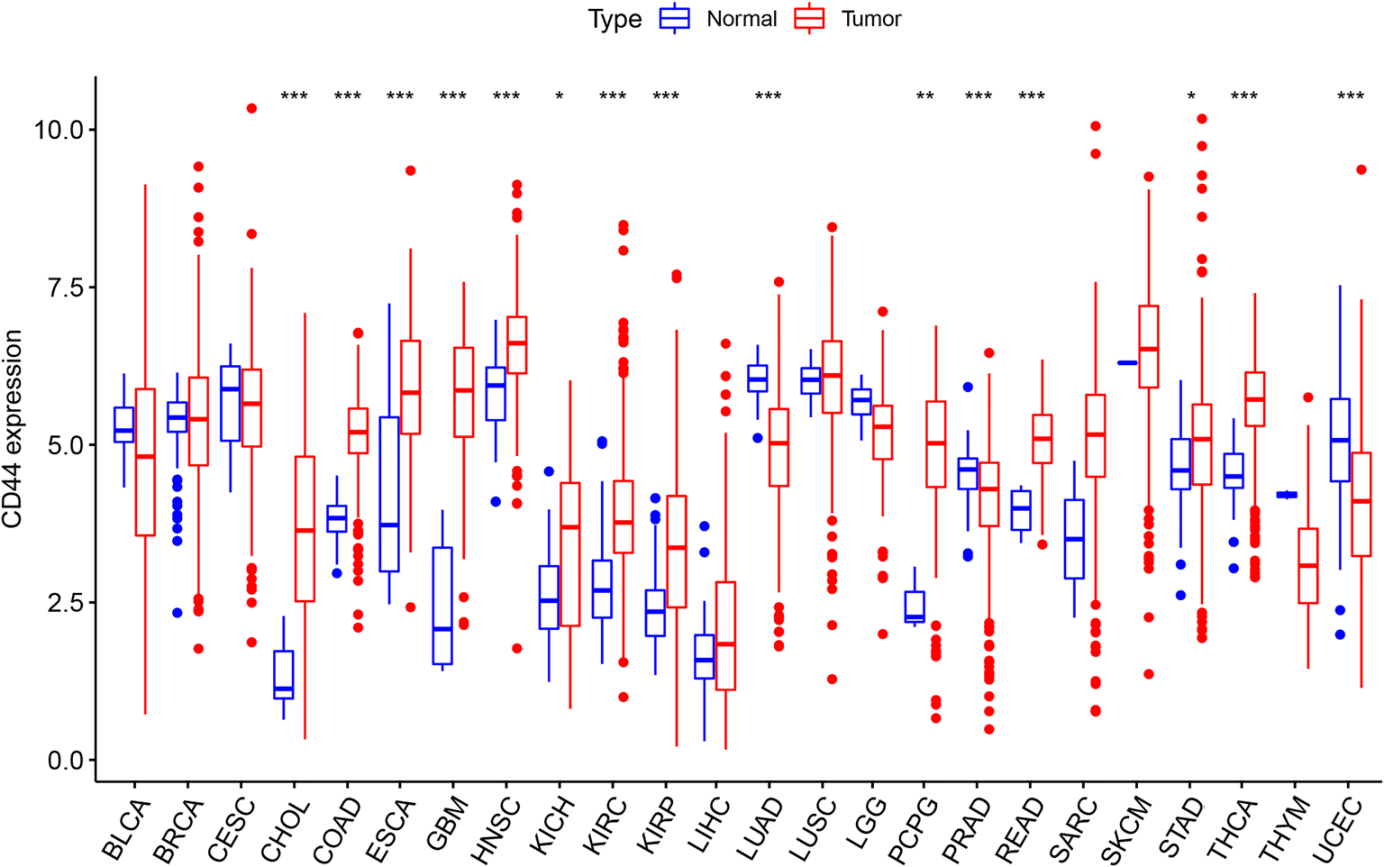
The expression level of CD44 in different cancers based on the TCGA database. *CD44* cluster of differentiation-44, *TCGA* The Cancer Genome Atlas. *P < 0.05, **P < 0.01, and ***P < 0.005.

To analyze the expression profiles of CD44 in the pan-cancer dataset, a comparative analysis of CD44 expression was performed between cancer and control samples using the TCGA database, the CD44 levels were significantly up-regulated in CHOL, COAD, ESCA, GBM, HNSC, KIRC, KIRP, READ, and THCA, whereas it was downregulated in LUAD, PRAD, and UCEC (Fig. 1). Collectively, these results revealed the difference in CD44 expression patterns between cancer and normal samples in the pan-cancer datasets.

### Correlation analysis between CD44 expression and survival

**Figure 2:**
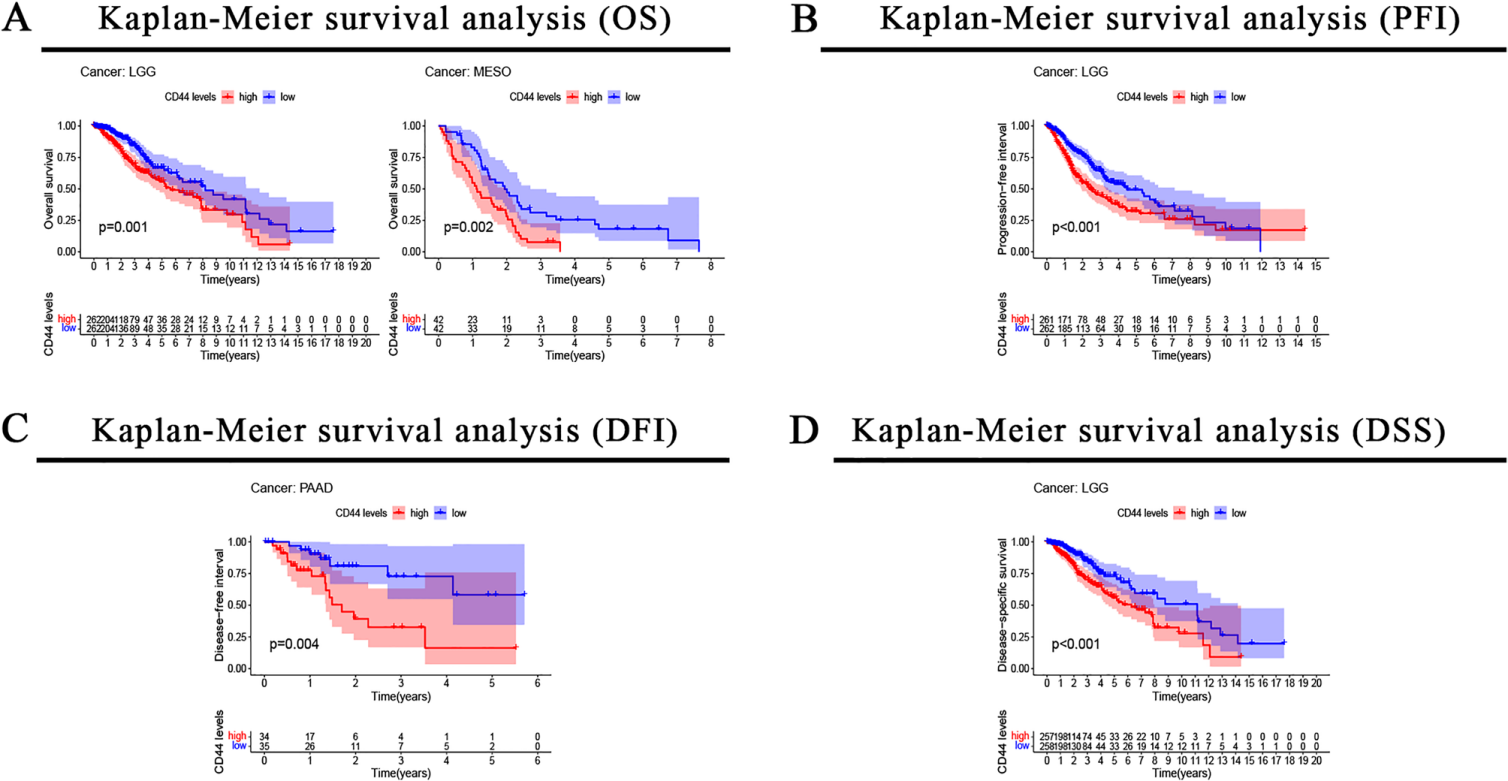
Correlation between CD44 expression level and OS (**A**), PFI (**B**), DFI (**C**), and DSS (**D**) as determined by Kaplan–Meier curve analyses. *OS* overall survival, *PFI* progression-free interval, *DFI* disease-free interval, *DSS* disease-specific survival.

Then, a correlation analysis of CD44 and the prognosis of pan-cancer patients were conducted. Survival indicators included OS, PFI, DFI, and DSS. In the OS analysis, the Kaplan–Meier survival curves indicated that the high expression of CD44 was remarkably associated with poor OS in LGG (*P* = 0.001), MESO (*P* = 0.002) (Fig. 2A). In the PFI analysis, Kaplan–Meier analysis showed that patients with higher CD44 expression had a shorter PFI in LGG (*P* < 0.001) (Fig. 2B). Likewise, results of Kaplan–Meier analysis indicated that patients with higher CD44 expression had a poorer DFI in PAAD (*P* = 0.004) (Fig. 2C). Besides, Kaplan–Meier analysis indicated that the increased CD44 expression correlated with poorer DSS in patients with LGG (*P* < 0.001) (Fig. 2D).

Cox regression of OS identified that CD44 expression was a risk factor for KIRC (*P* < 0.001), LGG (*P* < 0.001), PAAD (*P* < 0.001), however, it appeared to be a protective factor in UVM (*P* = 0.002) (see Supplementary Figure S1A online). Cox regression analysis of DSS demonstrated that CD44 expression as a risk factor in KIRC (*P* < 0.001), LGG (*P* < 0.001), PAAD (*P* = 0.004), while it was a protective factor in BRCA (*P* < 0.001), UVM (*P* = 0.002) (see Supplementary Fig. S1B online). Cox regression analysis of DFI noted that CD44 expression was a risk factor in PAAD (*P* = 0.001) (see Supplementary Fig. S1C online). Cox regression analysis of PFI identified that CD44 acted as a detrimental prognostic factor in KIRC (*P* < 0.001), LGG (*P* < 0.001), PAAD (*P* < 0.001) (see Supplementary Fig. S1D online) (Table 2). Altogether, these results signal that CD44 may serve as a prognostic biomarker and potential therapeutic target.

**Table 2:** Univariate Cox regression analysis of the associations of CD44 expression with patient survival.

Cancer	OS			PFI			DFI			DSS		
	HR	HR (95%CI)	*P*-value	HR	HR (95%CI)	*P*-value	HR	HR (95%CI)	*P*-value	HR	HR (95%CI)	*P*-value
ACC	0.964	(0.705–1.320)	0.821	0.874	(0.675–1.130)	0.303	0.977	(0.605–1.579)	0.925	0.889	(0.635–1.244)	0.492
BLCA	1.065	(0.966–1.173)	0.205	1.044	(0.945–1.153)	0.401	0.955	(0.756–1.206)	0.697	1.134	(1.004–1.281)	0.043
BRCA	0.924	(0.788–1.082)	0.324	0.817	(0.697–0.959)	0.013	0.798	(0.645–0.988)	0.038	0.700	(0.568–0.864)	< 0.001
CESC	1.069	(0.857–1.333)	0.555	1.005	(0.807–1.251)	0.965	0.782	(0.547–1.118)	0.177	1.012	(0.788–1.300)	0.924
CHOL	0.907	(0.668–1.232)	0.532	0.794	(0.598–1.055)	0.112	1.060	(0.731–1.538)	0.759	0.903	(0.652–1.249)	0.537
COAD	0.993	(0.736–1.339)	0.961	0.929	(0.701–1.232)	0.610	0.859	(0.467–1.577)	0.623	0.984	(0.673–1.439)	0.934
DLBC	0.800	(0.359–1.779)	0.584	0.795	(0.396–1.596)	0.519	0.950	(0.174–5.200)	0.953	1.068	(0.357–3.200)	0.906
ESCA	0.969	(0.765–1.226)	0.791	1.080	(0.876–1.331)	0.470	1.363	(0.912–2.039)	0.131	1.019	(0.772–1.344)	0.895
GBM	1.169	(0.990–1.380)	0.065	1.253	(1.054–1.489)	0.010	/	/	/	1.157	(0.965–1.386)	0.114
HNSC	1.176	(0.977–1.415)	0.086	1.236	(1.014–1.508)	0.036	1.191	(0.681–2.083)	0.539	1.321	(1.023–1.707)	0.033
KICH	1.051	(0.638–1.732)	0.844	1.050	(0.668–1.652)	0.831	0.772	(0.340–1.751)	0.535	1.136	(0.640–2.019)	0.663
KIRC	1.476	(1.269–1.718)	< 0.001	1.384	(1.182–1.620)	< 0.001	0.827	(0.485–1.411)	0.486	1.690	(1.408–2.030)	< 0.001
KIRP	1.289	(1.029–1.615)	0.027	1.112	(0.905–1.367)	0.312	1.073	(0.785–1.467)	0.657	1.363	(1.044–1.779)	0.023
LAML	0.779	(0.595–1.019)	0.068	/	/	/	/	/	/	/	/	/
LGG	1.416	(1.232–1.627)	< 0.001	1.318	(1.178–1.473)	< 0.001	1.223	(0.881–1.697)	0.229	1.481	(1.273–1.723)	< 0.001
LIHC	1.131	(0.984–1.300)	0.083	1.006	(0.891–1.136)	0.924	0.960	(0.834–1.105)	0.572	1.094	(0.908–1.319)	0.345
LUAD	1.021	(0.881–1.183)	0.782	1.004	(0.872–1.157)	0.952	1.011	(0.817–1.250)	0.923	1.006	(0.830–1.219)	0.953
LUSC	0.928	(0.796–1.083)	0.345	0.938	(0.778–1.133)	0.508	0.947	(0.706–1.269)	0.714	0.822	(0.653–1.034)	0.094
MESO	1.305	(1.033–1.649)	0.025	1.094	(0.863–1.386)	0.457	1.286	(0.700–2.361)	0.417	1.227	(0.919–1.639)	0.166
OV	0.895	(0.777–1.031)	0.125	0.882	(0.776–1.002)	0.054	0.817	(0.675–0.989)	0.038	0.869	(0.745–1.013)	0.073
PAAD	1.650	(1.245–2.188)	< 0.001	1.665	(1.257–2.206)	< 0.001	2.882	(1.508–5.505)	0.001	1.574	(1.154–2.147)	0.004
PCPG	0.780	(0.495–1.227)	0.282	0.723	(0.546–0.958)	0.024	0.716	(0.365–1.404)	0.331	0.742	(0.449–1.227)	0.245
PRAD	0.890	(0.504–1.572)	0.688	0.805	(0.655–0.991)	0.041	0.837	(0.575–1.218)	0.353	0.592	(0.272–1.285)	0.185
READ	0.628	(0.334–1.179)	0.147	0.843	(0.477–1.492)	0.559	0.682	(0.107–4.347)	0.685	1.047	(0.387–2.832)	0.928
SARC	0.828	(0.707–0.970)	0.019	0.943	(0.823–1.080)	0.394	0.961	(0.784–1.178)	0.702	0.801	(0.674–0.952)	0.012
SKCM	0.936	(0.827–1.059)	0.295	0.939	(0.844–1.045)	0.252	/	/	/	0.938	(0.820–1.073)	0.353
STAD	1.129	(0.978–1.304)	0.097	1.157	(0.992–1.348)	0.063	1.109	(0.836–1.472)	0.474	1.181	(0.986–1.413)	0.070
TGCT	2.256	(0.922–5.523)	0.075	0.926	(0.679–1.262)	0.626	0.871	(0.605–1.253)	0.457	2.226	(0.901–5.501)	0.083
THCA	0.724	(0.386–1.358)	0.314	1.122	(0.767–1.641)	0.552	1.343	(0.785–2.295)	0.282	0.492	(0.217–1.114)	0.089
THYM	1.674	(0.814–3.441)	0.161	1.697	(1.033–2.788)	0.037	/	/	/	1.157	(0.381–3.519)	0.797
UCEC	0.887	(0.740–1.062)	0.193	0.831	(0.711–0.972)	0.020	0.830	(0.662–1.042)	0.108	0.785	(0.628–0.981)	0.033
UCS	0.903	(0.646–1.262)	0.55	0.950	(0.701–1.289)	0.742	0.923	(0.500–1.705)	0.798	0.834	(0.578–1.203)	0.331
UVM	0.305	(0.143–0.650)	0.002	0.392	(0.194–0.794)	0.009	/	/	/	0.294	(0.136–0.637)	0.002

*OS* overall survival, *PFI* progression-free interval, *DFI* disease-free interval, *DSS* disease-specific survival.

### Correlation analysis between CD44 expression and pan-cancer clinicopathologic characteristics

Thereafter, the association between CD44 expression and clinicopathologic characteristics was investigated in pan-cancer datasets. In patients less than (or equal to) 65 years, a higher CD44 expression level was noted in ESCA, and UCEC. In contrast, CD44 was higher expressed in patients over 65 years old in LUAD (see Supplementary Figure S2A online). Moreover, CD44 was down-regulated during the advanced cancer clinical stage in BRCA, and SKCM (see Supplementary Fig. S2B online). Interestingly, CD44 expression also presented sex dimorphism in KIRC, and LUAD (see Supplementary Fig. S2C online), implying that CD44 may reflect clinical progression for those tumors.

### Correlation analysis of CD44 expression with TMB and MSI

TMB, MSI have been proposed to correlate with response to immunitherapy^24^, and we intend to evaluate the TMB and MSI status in CD44 expression to determine the potential of CD44 in reflecting the efficacy of immunotherapy to give suggestions on medication for cancer patients. High TMB was reported as a critical driver of cancer progression^25^. Our results determined a positive correlation between CD44 expression and TMB in COAD, LGG, and UCEC. In contrast, CD44 expression was negatively linked with TMB in BLCA, BRCA, ESCA, LUAD, LUSC, PRAD, and TGCT (see Supplementary Figure S3A online).

Meanwhile, MSI also acted as a predictive biomarker, enabling more precise guidance of immunotherapy^26^. Hence, the relationship of CD44 expression with MSI was analyzed. Our findings revealed a positive correlation between CD44 and MSI in COAD and UCEC. On the other hand, a negative correlation was discovered between CD44 and MSI in ESCA, HNSC, KIRC, and PRAD (see Supplementary Fig. S3B online). Next, we compared CD44’s predictive ability for immunotherapy efficacy to other canonical biomarker signatures in the TIDE database, using treatment responses from various cancer cohorts treated with ICB. The results confirmed that CD44 had a medium predictive performance, with 10 of the 25 ICB-treated cohorts presenting an area under curve (AUC) greater than 0.5 (see Supplementary Figure S4 online).

To sum up, these observations indicated that TMB and MSI were correlated in multiple cancer types, and the results provided by the TIDE database persuasively confirm its robustness in efficacy predictions, undoubtfully manifesting that it could be used as a reliable biomarker for predicting responses to immunotherapy.

### Associations between CD44 expression and therapeutic response of targeted therapy in various cancer

Targeted therapy and immunotherapy have become mainstream in cancer treatment. However, only some subsets of patients benefit from these therapies and more biomarkers needed to be explored. We investigated the utility of CD44 in evaluating therapeutic responses of targeted therapy to various cancer (see Supplementary Figure S5 online). In BRCA treated with anti-HER2 therapy, CD44 expression level was higher in non-responders, and with an AUC of 0.588. Likewise, CD44 expression was higher in non-responders in colorectal carcinoma treated with bevacizumab, with an AUC of 0.64. Furthermore, CD44 was associated with benefits of targeted therapeutic relapse-free survival (RFS) at 12 months in ovarian cancer, with an AUC of 0.733. Taken together, above results elucidated that CD44 could act as a therapeutic response biomarker in various cancer.

### Correlation between CD44 expression and various components in the TME of pan-cancer

**Figure 3:**
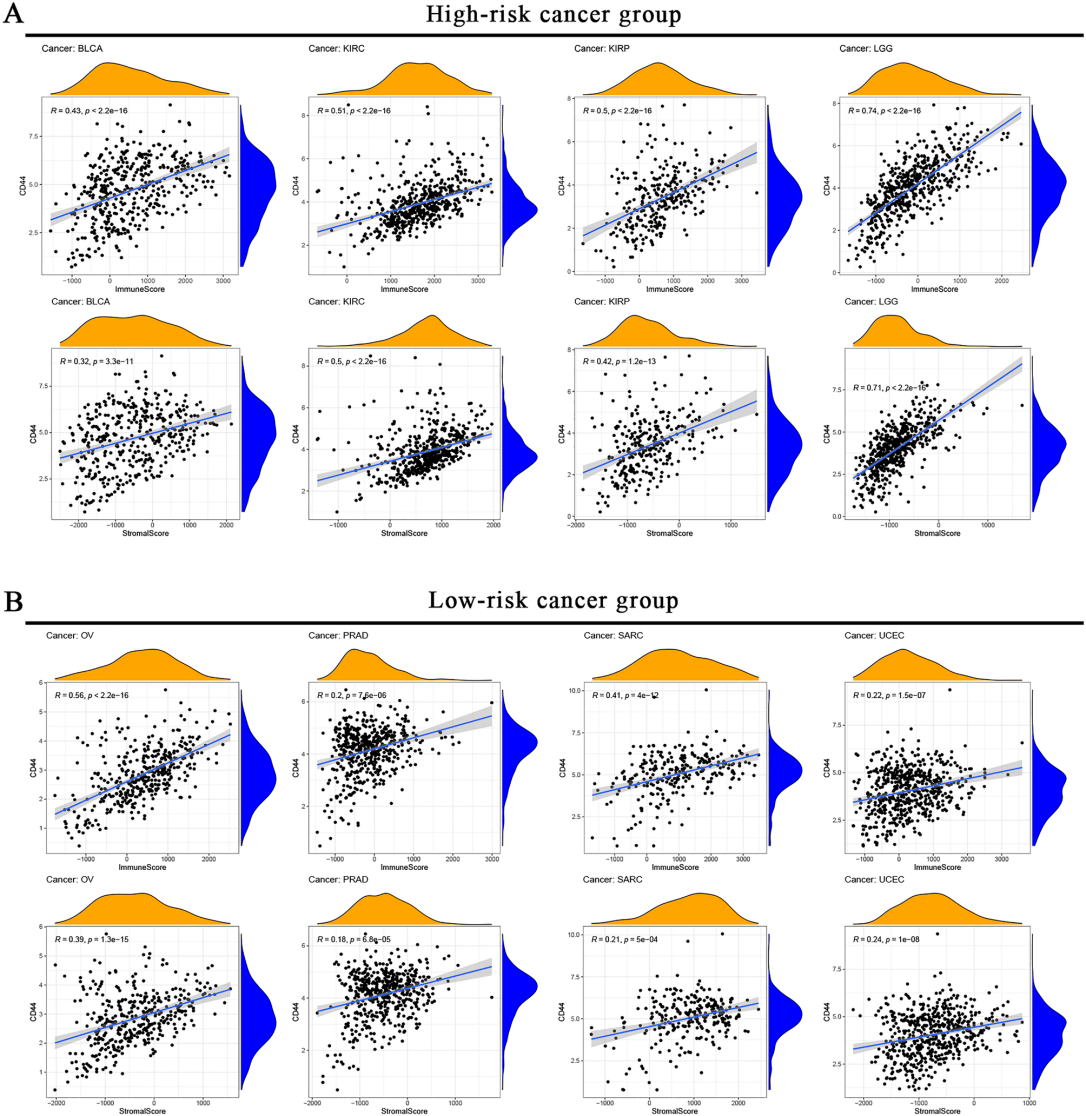
Correlation between CD44 expression and different components in the high-risk cancer group (**A**) and low-risk cancer group (**B**).

Components of the TME include tumor cells, stromal cells, and immune cells^27^, which can influence tumor formation, maintenance, and multidrug resistance, whereas non-malignant cells promote tumorigenesis in all stages of cancer^28^. Subsequently, the pan-cancer types were divided into high-risk cancer groups (BLCA, KIRC, KIRP, and LGG) (Fig. 3A) and low-risk cancer groups (OV, PRAD, SARC, and UCEC) (Fig. 3B) according to the survival outcome of the pan-cancer patients obtained from Kaplan–Meier curve and univariate Cox analysis. Unexpectedly, the immune score and stromal score were positively correlated with CD44 expression in every cancer type in both groups. Therefore, we hypothesized that CD44 could affect the immune and stromal components of TME.

**Figure 4:**
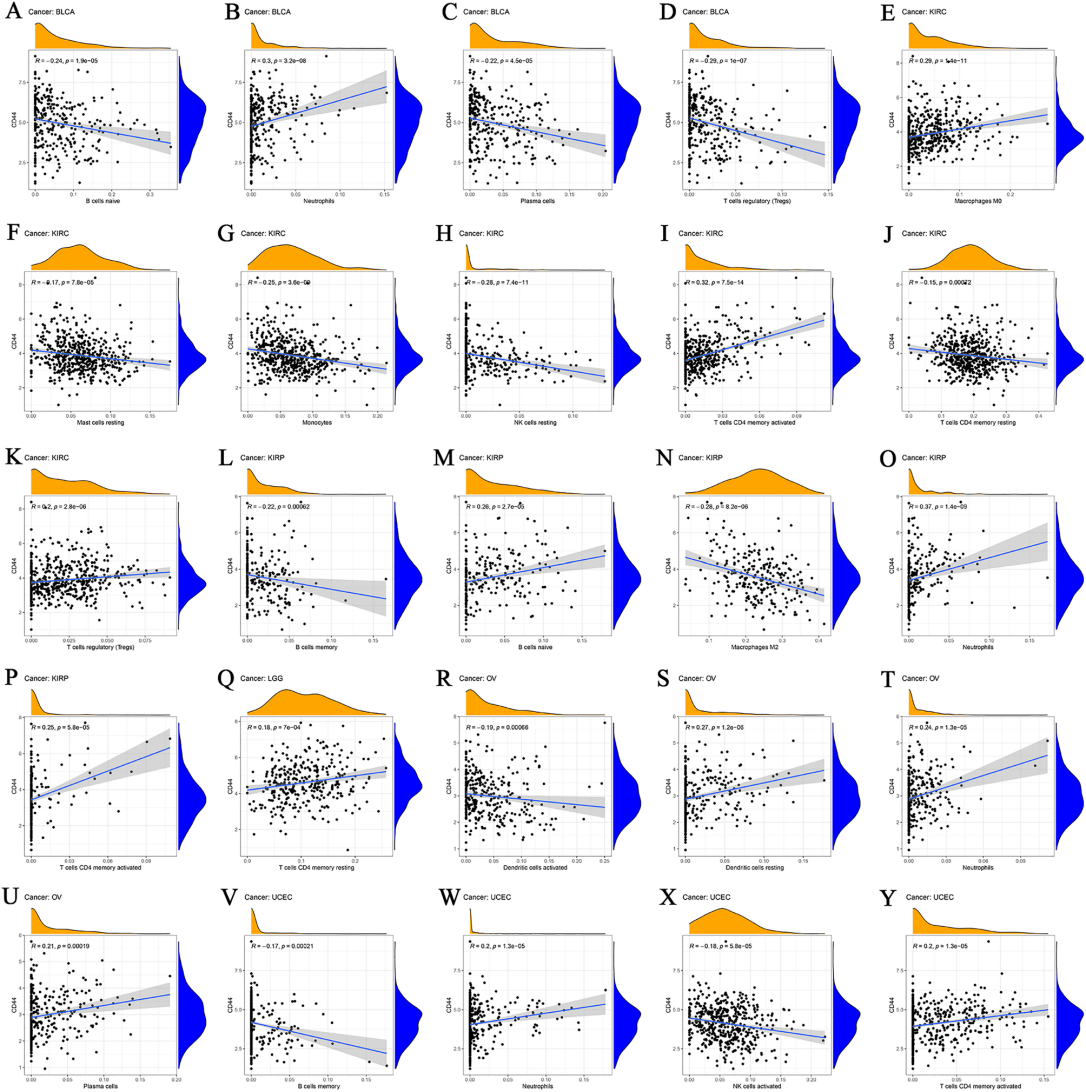
Correlation between the infiltrating status of immune cells with CD44 expression in BLCA (**A**–**D**), KIRC (**E**–**K**), KIRP (**L**–**P**), LGG (**Q**), OV (**R**–**U**), and UCEC (**V**–**Y**). *BLCA* bladder urothelial carcinoma, *KIRP* kidney renal papillary cell carcinoma, *LGG* brain lower grade glioma, *OV* ovarian serous cystadenocarcinoma.

**Figure 5:**
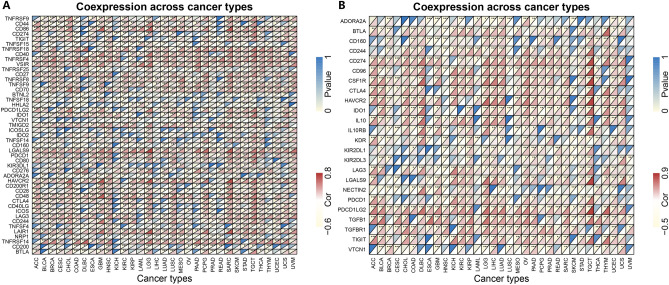
Correlation between CD44 expression and immune-related genes (**A**). Correlation between CD44 expression and immune checkpoint genes (**B**). *P < 0.05, **P < 0.01, and ***P < 0.005.

To further explore the association of CD44 expression and TIC subtypes, the CIBERSORT algorithm was utilized to calculate the relative levels of TIC subtypes in patients from both groups, with *P* < 0.001 as the cut-off value. It was observed that CD44 expression levels were positively linked with neutrophils in BLCA and negatively linked with naive B cells, plasma cells, and regulatory T cells (Tregs) (Fig. 4A–D). Moreover, CD44 expression was positively correlated with macrophages M0, activated memory CD4 T cells, and Tregs in KIRC, and negatively correlated with resting mast cells, monocytes, resting NK cells, and resting memory CD4 T cells (Fig. 4E–K). In addition, CD44 expression levels were significantly positively correlated with naive B cells, neutrophils, and activated memory CD4 T cells in KIRP but negatively correlated with memory B cells and macrophages M2 (Fig. 4L–P). Besides, a positive correlation between CD44 expression and resting memory CD4 T cells was noted in LGG (Fig. 4Q). In OV, CD44 expression levels were positively correlated with resting dendritic cells, neutrophils, and plasma cells and negatively correlated with activated dendritic cells (Fig. 4R–U). In UCEC, CD44 expression levels were positively correlated with neutrophils and activated memory CD4 T cells and negatively correlated with activated NK cells and memory B cells (Fig. 4V–Y). Collectively, these results infer that CD44 may mediate the immune response in these cancer types. Thereupon, correlation analysis was performed between CD44 and various immune-related genes (Fig. 5A), and immune checkpoint genes (Fig. 5B). Finally, the results suggested that CD44 may interfere with the TME by influencing the expression of immune-related genes and immune checkpoint genes, which mediates tumor progression and metastasis.

### Functional states of CD44

**Figure 6:**
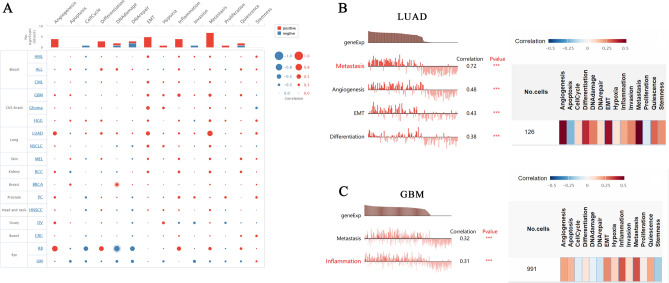
The correlation of CD44 with the functional state in cancers. The interactive bubble chart presents the correlation of CD44 with the functional state in 17 cancers (**A**). The correlation of CD44 with functional state in LUAD (**B**), GBM (**C**). *LUAD* lung adenocarcinoma, *GBM* glioblastoma multiforme. ∗∗∗*P* < 0.001.

Next, we explored the functional role of CD44 in TME of various cancer types using CancerSEA database, which shows the correlation of CD44 with malignant phenotype and functional states at single-cell resolutions. The results showed that CD44 expression had a positive correlation with the angiogenesis, differentiation, EMT, inflammation and metastasis (Fig. 6A). Then, we evaluated the correlation with CD44 and the functional status in specific cancers. The results elucidated that CD44 positively correlated with metastasis, angiogenesis, EMT, and differentiation in LUAD (Fig. 6B); with metastasis and inflammation in GBM (Fig. 6C). Therefore, we tentatively proposed that CD44 may promote malignant phenotypes of cancer cells, which could thus be used as a potential therapeutic target for some specific cancer types.

### Cancers enrichment analysis

**Figure 7:**
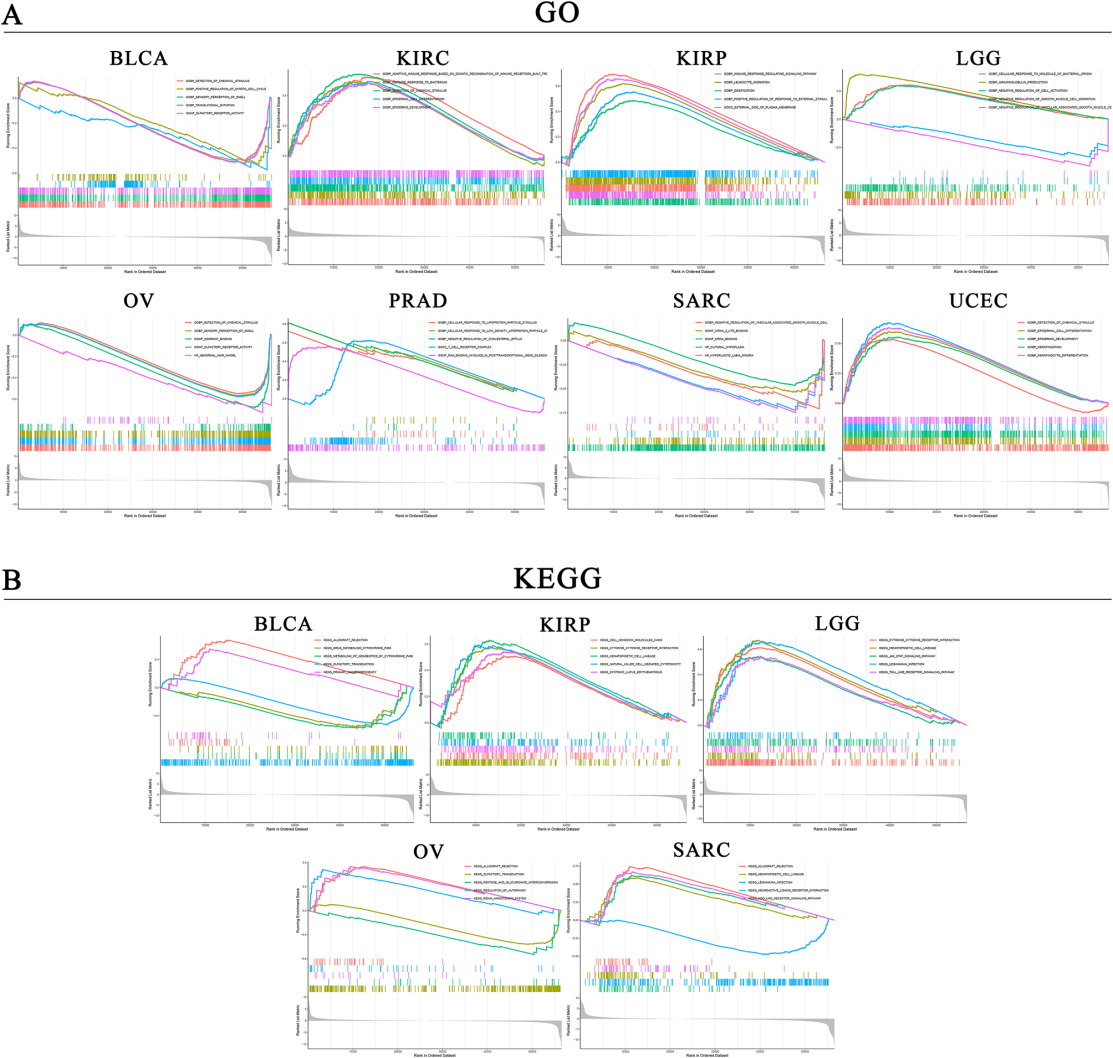
GO functional terms (**A**) and KEGG pathway analysis (**B**) of CD44 in various cancer types in the high- and low-risk cancer groups. *GO* Gene ontology, *KEGG* Kyoto Encyclopedia of Genes and Genomes.

To elucidate the underlying molecular mechanism of CD44 in tumorigenesis, GSEA was performed to assess the biological significance of CD44 expression in eight pan-cancer types (Fig. 7). In GO functional annotation, CD44 was significantly correlated with several immune-related functions in KIRC, KIRP, and UCEC, such as leukocyte migration and detection of chemical stimulus (Fig. 7A). Furthermore, KEGG analyses demonstrated that CD44 could positively influence several crucial immune cell-related pathways in KIRP and LGG, such as the toll-like receptor signaling pathway and Leishmania infection (Fig. 7B). Overall, these results confirmed that CD44 is instrumental in TME remodeling for various cancers.

## Discussion

The pan-cancer analysis can disclose the heterogeneities of tumors, providing insights into cancer treatment^29^. Numerous pan-cancer studies have focused on gene mutations and cancer development, which are helpful in the progression of sustainable, meaningful clinical treatments and the development of biomarkers^30^. As previously reported, CD44 is overexpressed in CSCs and plays a vital role in cancer progression, metastasis, and drug resistance^31^. It may also serve as a therapeutic target, given that it modulates multiple survival signaling pathways^10,32^. It has been previously reported that CD44 can promote CSC traits of metastatic breast cancers by activating the PDGFRβ/Stat3 signaling pathway^11^. Accumulating evidence revealed that CD44 might present as a therapeutic biomarker in various tumor types^12,33^. Although CD44 has been extensively studied in certain types of cancer, its role remains elusive in multiple cancers. In this research, we described the functional significance of CD44 and identified the differential expression of CD44 within cancers and normal tissues in 12 cancer types from the pan-cancer datasets. Moreover, we also confirmed that CD44 expression was relevant to the levels of immune cell infiltration in various types of cancer with ESTIMATE and CIBERSORT. Lastly, GSEA analysis exposed that CD44 was significantly correlated with several signaling pathways.

CSCs are hypothesized to possess the ability of self-renewal, tumor initiation and metastasis. Prior research reported that the overexpression of CD44 in cancer cells is widely accepted as a marker of higher tumor-initiating potential and invasiveness of cancer cells^34,35^. CD44 is recognized as the CSC surface marker for sorting cancer types such as breast cancer^9^, prostate cancer^36^, and gastric cancers^16^. Previous studies revealed that CD44 might be unnaturally expressed in several cancer types and play an essential role in cancer progression. Herein, significant upregulation of CD44 expression levels was observed in cancer tissues compared to normal tissues in CHOL, COAD, ESCA, GBM, HNSC, KIRC, KIRP, READ, and THCA, while down-regulated in LUAD, PRAD, and UCEC. Interestingly, some studies reported contrasting outcomes. For instance, a study reported that CD44 was up-regulated in LUAD, showed significantly higher capacities of tumorigenic colonies^37^, and was related to worse OS^38^. Notably, the functional role of CD44 on cancer development and progression has become a research hotspot and will assist in understanding its potential role as a prognostic biomarker for cancers.

Furthermore, our results established that a higher CD44 expression level was related to unfavorable survival outcomes in LGG, MESO, and PAAD. Similar outcomes were also observed in glioma patients^39^. Compelling evidence obtained from 42 studies outlined that gastric cancer patients with CD44 overexpression had a lower 5-year OS rate^40^. Some studies also reported similar results in colorectal cancer^41,42^. Moreover, overexpression of CD44 predicted a poor prognosis in patients with hepatocellular carcinoma^43^ and pancreatic carcinoma^44^. Besides, another study revealed that expression of CD44 varied significantly by age and gender in oral cancer^45^, which is consistent with the outcomes of this study, where CD44 was up-regulated in older patients with LUAD and downregulated in older patients affected by ESCA, and UCEC. Moreover, some studies revealed a novel potential therapeutic target that survival outcomes are also affected by stem cells, which can be regulated by stemness-related genes^46,47^. In short, these outcomes strongly indicate that CD44 might be a useful biomarker for most cancer types.

Gene mutation is postulated to be the primary cause of cancer^48^, and specific gene mutations have distinct impacts on the prognosis and risk stratification of various cancer types^49^. TMB is defined as the number of somatic mutations per megabase of the interrogated genomic sequence, while MSI is defined as the collection of microsatellite mutations; both are widely used as predictive biomarkers of response to immunotherapy^50,51^. Additionally, recent studies have demonstrated that MSI and TMB contribute significantly to the therapeutic response to immune checkpoint inhibitors (ICIs)^52^. The MSI-low phenotype was found as a worse prognostic biomarker in colorectal cancers^53^. However, MSI has limitations, such as immune checkpoint blockade failing to elicit a response in colorectal cancer cases^54^. This research established a relationship between CD44 and TMB and MSI, implying that CD44 may provide a more comprehensive perspective of immunotherapy in these cancer types.

The MSI status may alter the TME of cancer patients, thereby affecting the efficacy of ICIs^55^, while TME plays a crucial role in tumorigenesis and cancer progression^56,57^. Increasing evidence indicated that the immune escape of cancer cells is correlated with various components of the TME and ultimately contributes to tumor proliferation, metastasis, and recurrence. And the effect of risk scores on the TME may have essential roles in cancer development^58^. Albeit immunotherapy has made considerable advances in cancer treatment, it still faces numerous challenges in its successful application^59,60^. Indeed, to further improve the efficacy of immunotherapy, the identification of novel biomarkers is vital. Gomez et al. reported that CD44 expression was regulated by TAM, which directly influences CD44 signaling via ligand binding in HNSC^12^. Nonetheless, little is known about the role of CD44 in the immune microenvironment. The results from this study indicated that CD44 level was significantly correlated with T cells, B cells, NK cells, macrophages, and other immune infiltrating cells in BLCA, KIRC, KIRP, LGG, OV, and UCEC. Taken together, it is reasonable to speculate that CD44 may play an essential role in cancer immunity and ultimately influence prognosis. This study still has some limitations, biological validation and large sample cancer cohort validation should be performed to better illustrate the role of CD44 in the pan-cancer study.

## Conclusions

In summary, our results indicated that CD44 was associated with disease prognosis and immune infiltration in pan-cancers. Moreover, CD44 expression was also linked with TMB, MSI, and various components of the TME. These findings add to the understanding of tumor mechanisms and contribute to improving the efficacy of immunotherapy.

## Supplementary Information

Supplementary Information 1.

Supplementary Information 2.

Supplementary Information 3.

Supplementary Information 4.

Supplementary Information 5.

Supplementary Information 6.

## Data Availability

The datasets supporting the conclusions of this article are available in the University of California Santa Cruz Xena (UCSC) Xena repository (http://xena.ucsc.edu/).
