# Supplementary material for: The prognostic value and immunological role of CD44 in pan-cancer study
Source: Sci Rep. 2023 Apr 28;13:7011. doi: 10.1038/s41598-023-34154-3 (PMC10147611; doi:10.1038/s41598-023-34154-3)
Supplement: Supplementary file 1 — Supplementary Information 1. [file 41598_2023_34154_MOESM1_ESM.docx]

**Figure S1** Univariate Cox analysis of the correlation between CD44 expression level and survival data.

**Notes: A:** Overall survival (OS); **B:** Progression free interval (PFI); **C:** Disease free interval (DFI); **D:** Disease specific survival (DSS).

**Figure S2** The correlation of CD44 expression with clinical annotations.

**Notes:** CD44 expression level was correlated with age in ESCA, LUAD, and UCEC (A), with tumor stages in BRCA, and SKCM (B), with gender in KIRC, and LUAD (C).

**Abbreviations:** ESCA: esophageal carcinoma, LUAD: lung adenocarcinoma, THYM: thymoma, UCEC: uterine corpus endometrial carcinoma, BRCA: invasive breast carcinoma, COAD: colon adenocarcinoma, KIRC: kidney renal clear cell carcinoma, SKCM: cutaneous skin melanoma, LIHC: liver hepatocellular carcinoma.

**Figure S3** Relationships between CD44 expression and TMB, MSI.

**Notes:** Relationships between CD44 expression and TMB (A), MSI (B) in pan-cancer patients.

**Abbreviations:** TMB: tumor mutational burden, MSI: microsatellite instability.

∗*P* < 0.05, ∗∗*P* < 0.01, and ∗∗∗*P* < 0.005.

**Figure S4** The potential of CD44 as a responsive biomarker in the TIDE database.

**Notes:** Bar plot presenting the predictive ability of CD44 for immunotherapy efficacy compared with other canonical biomarker signatures, based on treatment response, and prognosis of varying cancer cohorts treated with immune checkpoint blockade (ICB) in TIDE database.

**Figure S5** Associations between the CD44 expression levels and therapeutic responses in various cancer cohorts.
