# Supplementary figures and images for: The prognostic value and immunological role of CD44 in pan-cancer study
Source: Sci Rep. 2023 Apr 28;13:7011. doi: 10.1038/s41598-023-34154-3 (PMC10147611; doi:10.1038/s41598-023-34154-3)

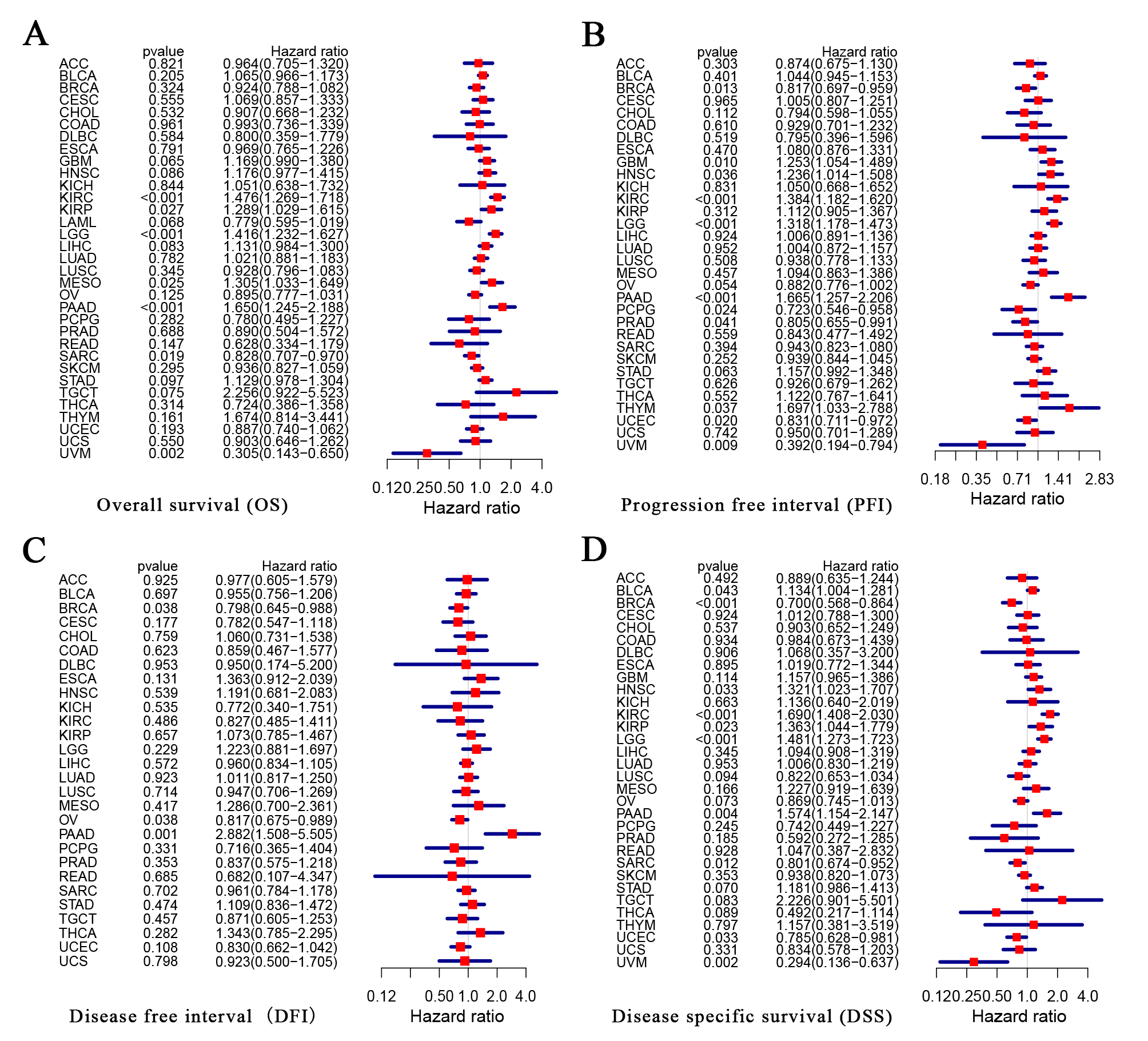

Supplement: Supplementary file 2 — Supplementary Information 2. [file 41598_2023_34154_MOESM2_ESM.tif]

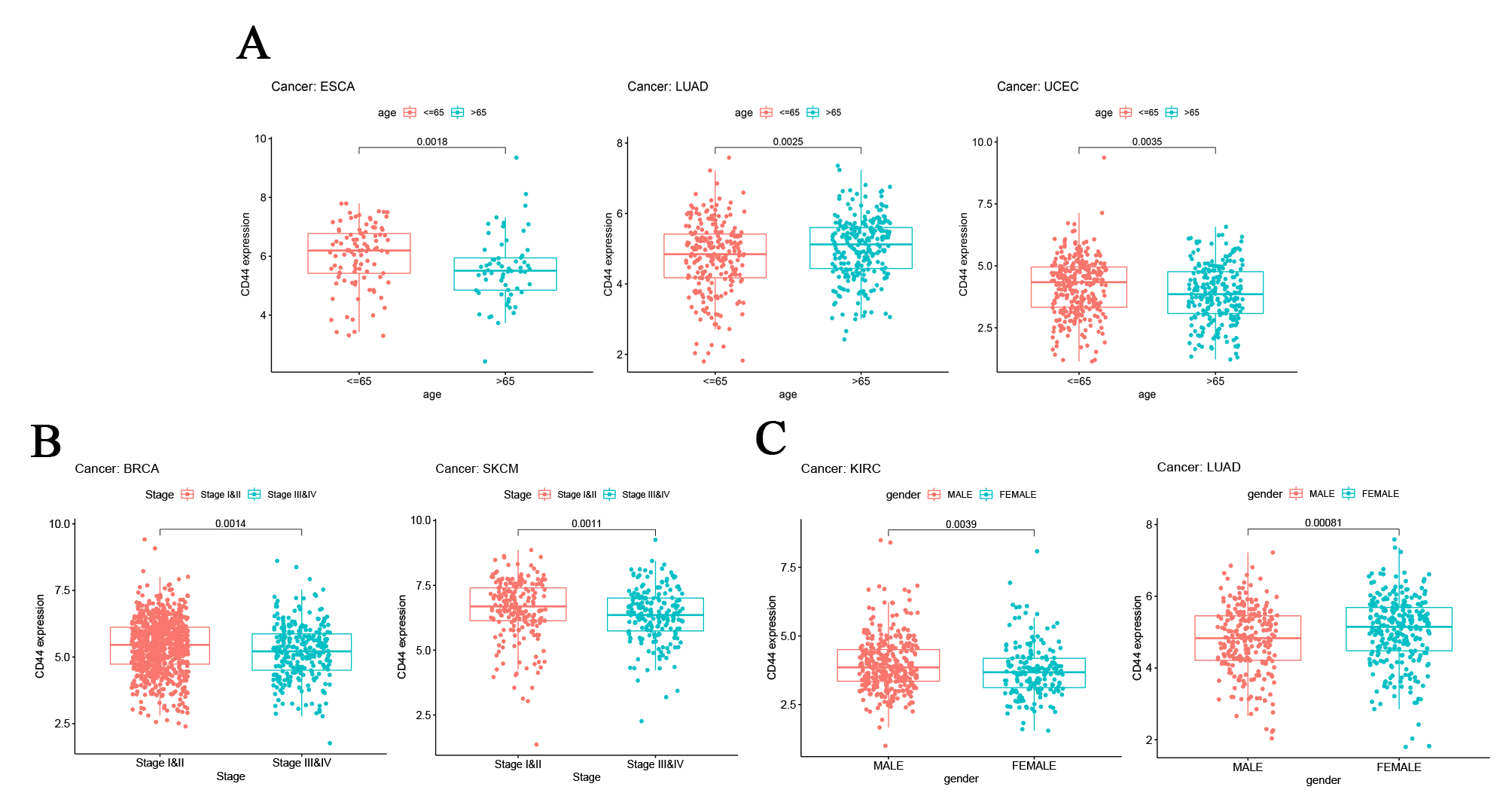

Supplement: Supplementary file 3 — Supplementary Information 3. [file 41598_2023_34154_MOESM3_ESM.tif]

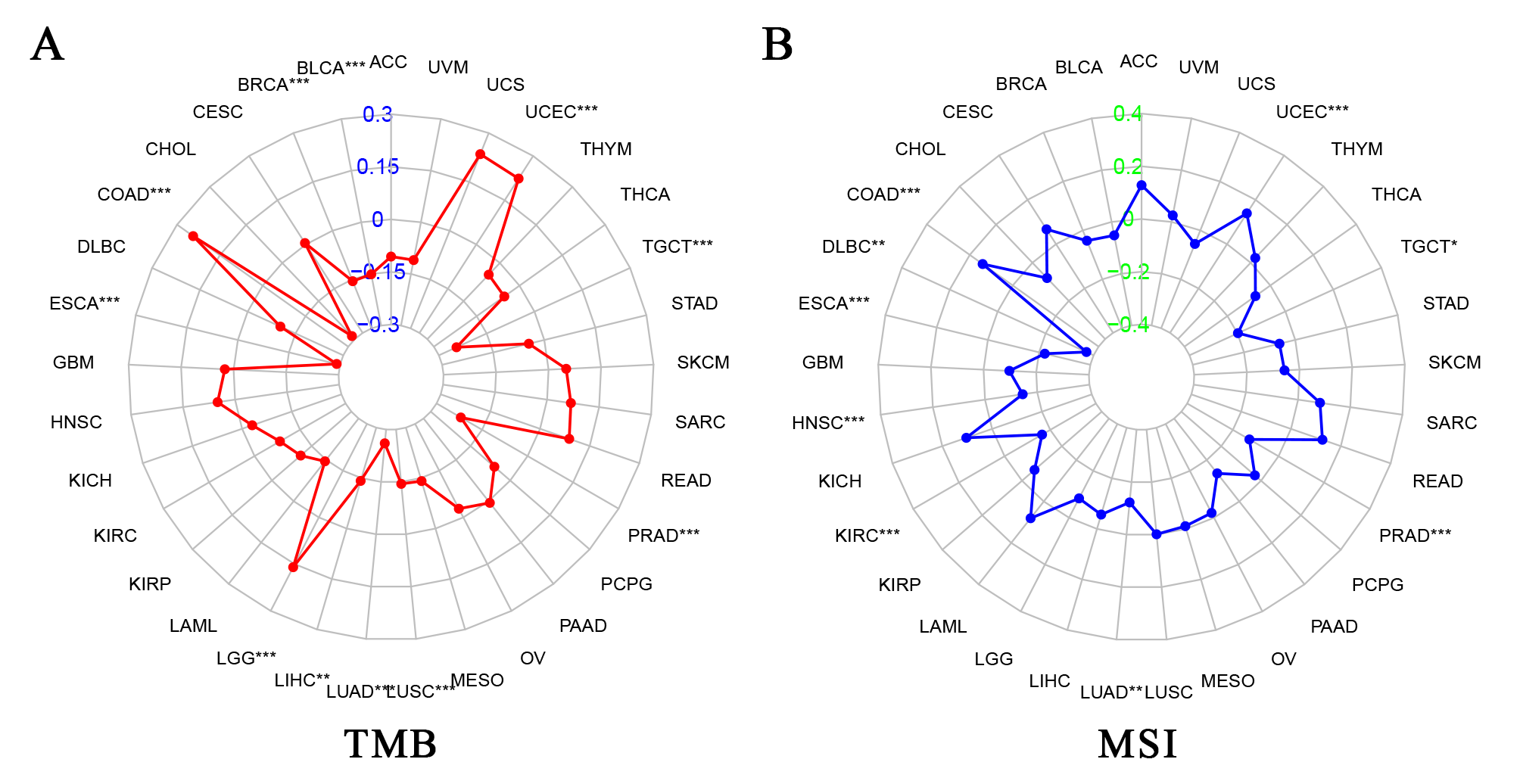

Supplement: Supplementary file 4 — Supplementary Information 4. [file 41598_2023_34154_MOESM4_ESM.tif]

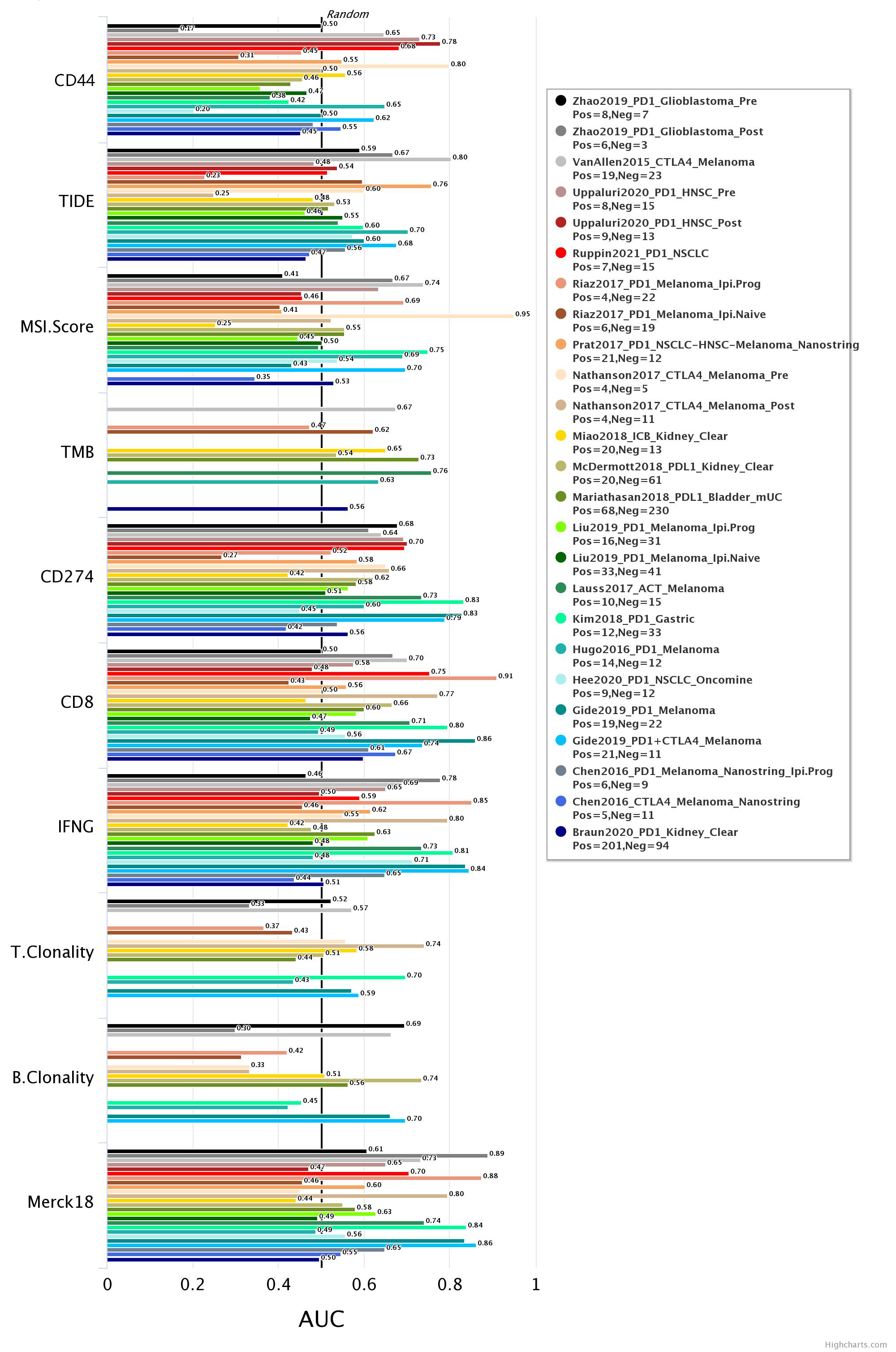

Supplement: Supplementary file 5 — Supplementary Information 5. [file 41598_2023_34154_MOESM5_ESM.tif]

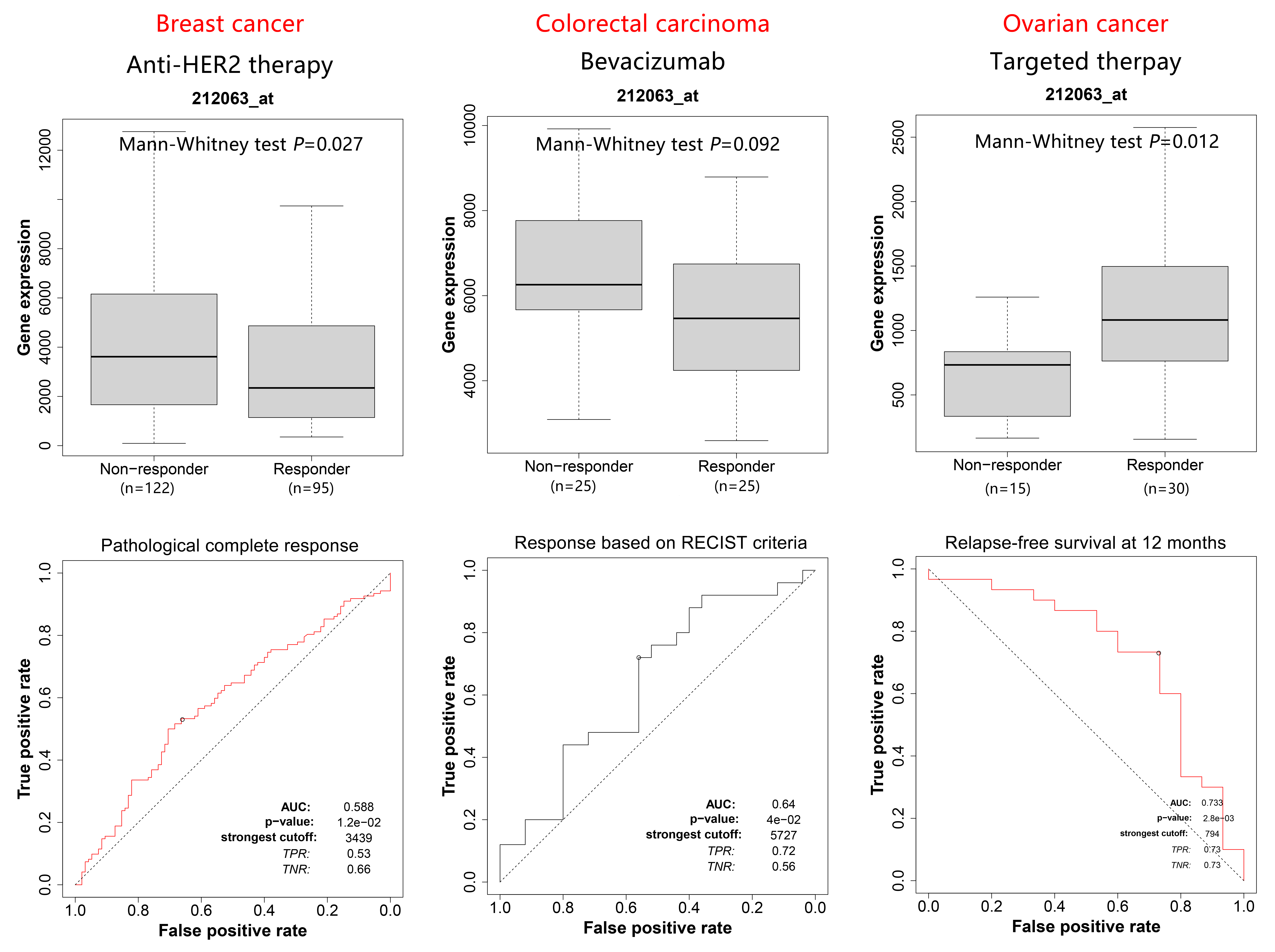

Supplement: Supplementary file 6 — Supplementary Information 6. [file 41598_2023_34154_MOESM6_ESM.tif]
